# Treatment Strategy of Metastatic Nasopharyngeal Carcinoma With Bone Marrow Involvement—A Case Report

**DOI:** 10.3389/fonc.2022.877451

**Published:** 2022-06-07

**Authors:** Bicheng Zhang, Ting Zhang, Lan Jin, Yan Zhang, Qichun Wei

**Affiliations:** ^1^ Department of Radiation Oncology, The Second Affiliated Hospital of Zhejiang University, School of Medicine, Hangzhou, China; ^2^ Department of Laboratory Medicine, The Second Affiliated Hospital of Zhejiang University, School of Medicine, Hangzhou, China; ^3^ Department of Medical Oncology, The Second Affiliated Hospital of Zhejiang University, School of Medicine, Hangzhou, China

**Keywords:** bone marrow involvement, cetuximab, nasopahryngeal carcinoma, patient-derived tumor xenograft (PDTX), long-term survival

## Abstract

Bone marrow involvement (BMI) of solid tumors is a special type of distant metastasis. It has an occult onset, atypical clinical and laboratory features, and a high mortality. We present a nasopharyngeal carcinoma case with cervical and axillary lymph nodes, bilateral lung, multiple bone, and bone marrow metastases, who was treated chemotherapy plus targeted therapy under the guidance of a patient-derived tumor xenograft (PDTX) model, followed by maintenance chemotherapy plus immunotherapy. The patient’s symptoms were relieved after four cycles of chemotherapy plus targeted therapy. His bone marrow biopsy turned negative after 7 months of therapy. In addition, his total peripheral T cells as well as the proportion of CD8^+^ T cells increased during the course of therapy. The combination of chemotherapy, targeted therapy, and immunotherapy provides an effective antitumor regimen for advanced NPC patients with BMI.

## Introduction

Bone marrow involvement (BMI) in solid tumors is rare in clinical practice compared with that of hematological tumors. The incidence rate of BMI in solid tumors is reported to range from 11% to 20% (1–4), which is increased to 23%–35% in recurrent or metastatic tumors (4–8). The BMI in solid tumors usually indicates a poor prognosis. The median overall survival time for solid tumor patients with BMI was reported to be 49 days (9). However, some case reports have confirmed that systematic treatment can prolong the survival rate of patients with BMI (10–15). There is no standardized treatment for patients with BMI in solid tumors because of the lack of clinical trial data.

The incidence rate of BMI in nasopharyngeal carcinoma (NPC) is unclear. Berry reported a 63-year-old man with NPC and BMI who died within 2 weeks of the diagnosis without antitumor therapy (16). Miyaushiro reported a 51-year-old man with NPC and BMI who maintained a good performance status for 7 months during weekly paclitaxel chemotherapy but died from multiple organ failure 8 months after diagnosis (17). Here, we present a metastatic NPC patient with BMI who had long-term survival after chemotherapy plus anti-EGFR-targeted therapy according to a PDTX model, followed by maintenance therapy with chemotherapy plus immunotherapy according to the peripheral immune environment.

## Case

**Figure 1:**
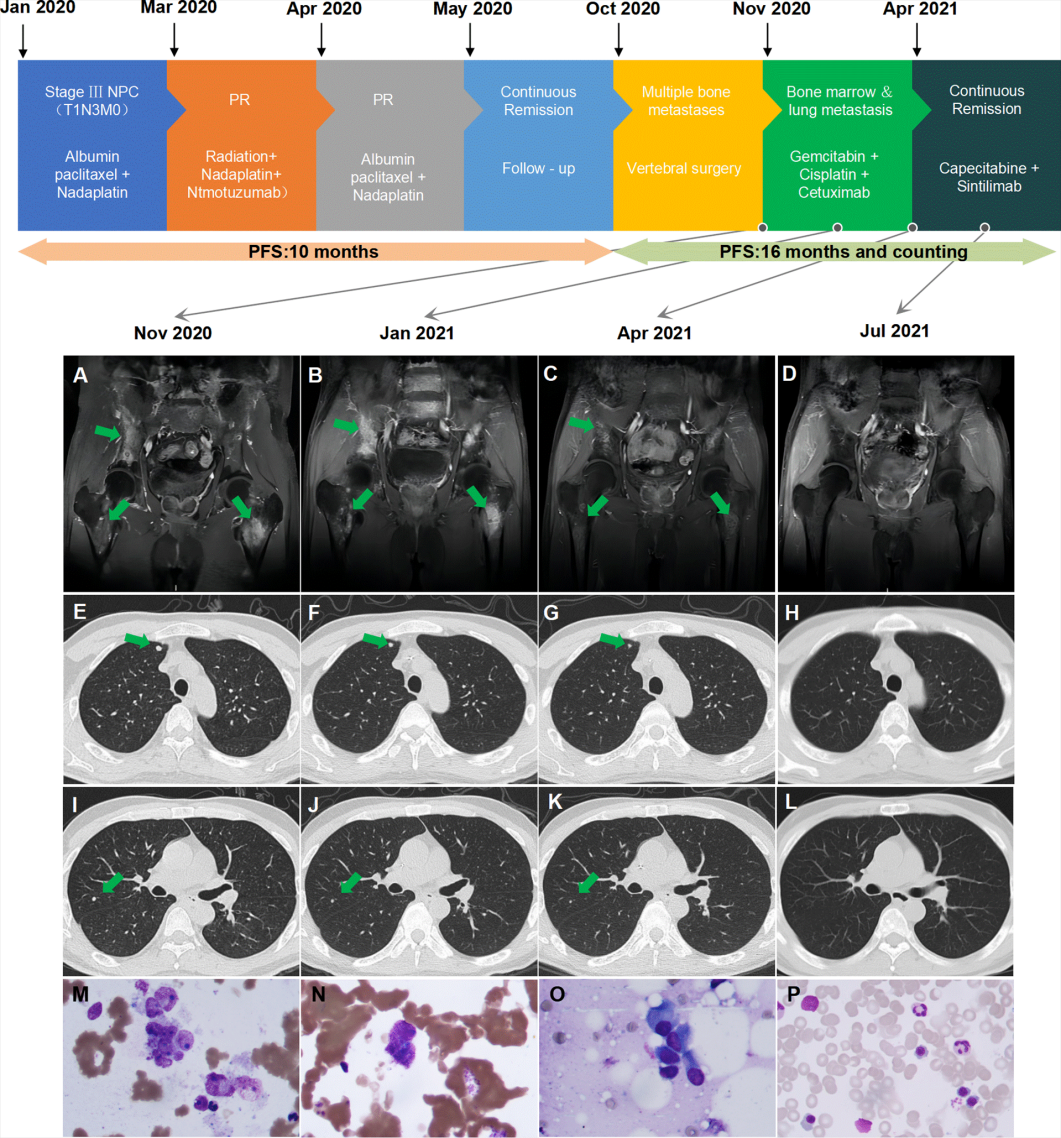
Summary of the patient’s treatment history. **(A)** Multiple bone metastases before second-line treatment. **(B)** Multiple bone metastases after two cycles of therapy. **(C)** Reduction of multiple bone metastases after six cycles of therapy. **(D)** Continued reduction of multiple bone metastases after 3 months of maintenance therapy. **(E, I)** Multiple lung metastases before second-line treatment. **(F, J)** Multiple lung metastases after two cycles of therapy. **(G, K)** PR of multiple lung metastases after six cycles of therapy. **(H**, **L)** Disappearance of lung metastases after 3 months of maintenance therapy. **(M)** Clustered tumor cells confirmed bone marrow involvement before second-line treatment. **(N)** Scattered tumor cells detected after four cycles of therapy. **(O)** Suspicious tumor cells detected after six cycles of therapy. **(P)** Bone marrow aspiration negative after 3 months of maintenance therapy. PR, partial response; PFS, progression-free survival.

A 44-year-old man was initially diagnosed in our center with stage IVa (T1N3M0, American Joint Committee on Cancer (AJCC) 8th edition) NPC and dermatomyositis in December 2019. Positron emission tomography–computed tomography (PET-CT) showed the primary tumor was confined to the nasopharynx, accompanied by lymph node metastasis in the left neck, left parapharyngeal space, and left clavicle. The diagnostic and treatment history of the patient is shown in **Figure 1**. Three cycles of induction chemotherapy with paclitaxel-albumin (240 mg/m^2^, every 3 weeks) plus nedaplatin (90 mg/m^2^, every 3 weeks) were conducted from January 4, 2020, to March 4, 2020. Partial remission was achieved after induction chemotherapy, assessed by the nasopharyngeal magnetic resonance imaging (MRI) and cervical CT scan, according to the Response Evaluation Criteria in Solid Tumors (RECIST) 1.1. Intensity-modulated radiotherapy (70 Gy/32 fractions) with two cycles of nedaplatin (90 mg/m^2^, every 3 weeks) concurrent chemotherapy and fixed-dose weekly nituzumab (200 mg) concurrent anti-EGFR-targeted therapy was conducted from March 13, 2020, to April 28, 2020. The patient received one cycle of adjuvant chemotherapy of paclitaxel-albumin plus nedaplatin. Complete remission was achieved in the imaging evaluation 1 month after the completion of radiotherapy. From then on, he had regular visits until August 2020, when no recurrence and metastasis were detected.

The patient suffered from lumbar pain on October 9, 2020. MRI and PET-CT scans revealed right scapula, right 3rd rib, left 6th rib, thoracic 10 to lumbar 5 vertebral bodies and some accessories, sacrum, bilateral iliac bones, right ischium, left upper femur metastases, and lung metastases. Subsequently, he underwent posterior thoracolumbar tumor resection, reconstruction, and internal fixation under general anesthesia on October 27, 2020. The postoperative pathology was metastatic nonkeratinized undifferentiated carcinoma, which was confirmed as a bone metastasis of NPC. PDTX models were built using cancer cells obtained from surgery to evaluate the sensitivity of different regimens of systemic therapy.

**Figure 2:**
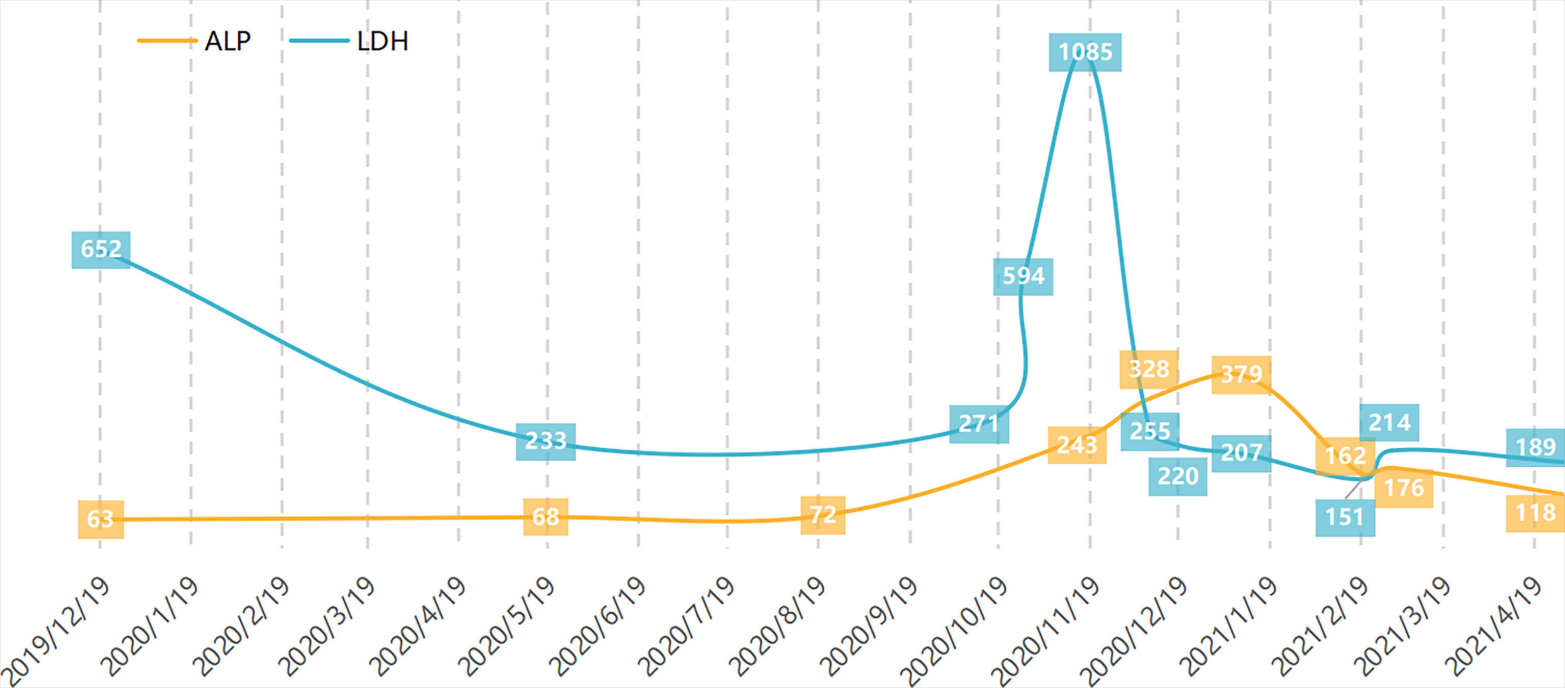
Alkaline phosphatase and lactase dehydrogenase changes during treatment (U/L).

He was readmitted on November 17, 2020. At that time, he had a Karnofsky performance status (KPS) score of 50, a continued fever of more than 39°C, and lumbar pain, which caused him to be bedridden for 3 weeks after the operation. MRI and PET-CT scans revealed that he had rapid progression with bone and lung metastasis. BMI was detected from bone marrow aspiration and biopsy (**Figure 1M**). Whole blood counts from the blood test indicated hemoglobin (HB) of 111 g/L (normal range: 131–172 g/L), platelet count (PLT) of 339 10^9^/L (normal range: 100–300 10^9^/L), glutamic-pyruvic transaminase (GPT) of 85 U/L (normal range: 9–50 U/L), glutamic oxaloacetic transaminase (GOT) of 99 U/L (normal range: 15–40 U/L), alkaline phosphatase (ALP) of 243 U/L (normal range: 30–120 U/L), lactate dehydrogenase (LDH) of 1,085 U/L (normal range: 120–250 U/L), and C-reactive protein (CRP) of 134.9 mg/L (normal range: <10 mg/L). He had no liver metastasis or chronic liver disease, and dermatomyositis was controlled in a stable state. Based on his general situation and the result of the PDTX model, he was administered gemcitabine (1 g/m^2^, days 1 and 8, every 3 weeks) plus cetuximab (250 mg/m^2^, weekly) from November 29, 2020. After two cycles of treatment, the patient’s temperature returned to normal (below 37.4°C), the pain eased, the KPS score increased to 80, and partial remission was found in all lymph nodes, lung, and bone lesions at the first assessment after two cycles of treatment. Cisplatin (50 mg/m^2^, weekly) was added to the regimen beginning with the 3rd cycle of therapy. Repeated MRI and CT scans showed continued partial remission after four and six cycles of treatment. ALP and LDH levels continuously declined. The dynamic changes of ALP and LDH over the treatment are exhibited in **Figure 2**. Grade III leukocytopenia and thrombocytopenia were observed after chemotherapy and recovered after treatment with granulocyte colony-stimulating factor (G-CSF) and thrombopoietin (TPO), without chemotherapy delay.

The patient was then administered capecitabine plus sintilimab as maintenance therapy on April 9, 2021. Metastatic tumor cells could still be detected from bone marrow biopsy after 4 and 6 cycles of treatment on February 19, 2021, and April 9, 2021 (**Figures 1N, O**). Eventually, his bone marrow biopsy turned negative after 3 months of capecitabine plus sintilimab maintenance chemotherapy and immunotherapy on July 28, 2021 (**Figure 1P**). As of press date, he has survived for 16 months since bone marrow involvement. He completed 15 cycles of capecitabine plus sintilimab with a KPS of 100 and remains in continuous remission every 3 months of assessment. However, he refused further bone marrow aspiration.

**Figure 3:**
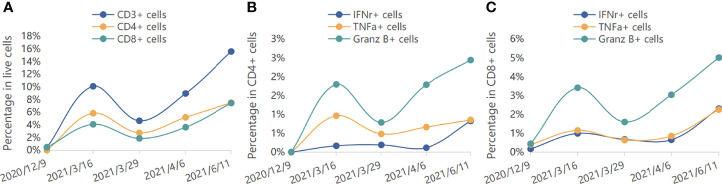
Reconstitution kinetics in immune cells in the peripheral blood. **(A)** The percentages of CD3^+^, CD4^+^, and CD8^+^ cells in live cells during treatment. **(B)** The percentages of IFNr^+^, TNFa^+^, and Granz B^+^ cells in CD4^+^ cells during treatment. **(C)** The percentages of IFNr^+^, TNFa^+^, and Granz B^+^ cells in CD8^+^ cells during treatment.

Reconstitution kinetics in immune cells in the peripheral blood during the period of maintenance treatment is shown in **Figure 3**. We found that the proportion of total peripheral T cells as well as the proportion of CD8^+^ T cells increased. Intracellular staining showed that after combined immunotherapy, peripheral CD8^+^ T cells were transformed into activated T cells, which express high levels of TNF-α, IFN-γ, and granzyme B. The production of TNF-α and IFN-γ in CD4^+^ T cells was also increased.

## Discussion

Here, we presented a successful case of a metastatic NPC patient with BMI who had long-term survival and was effective in antitumor treatment effects. A complete response on bone marrow biopsy was shown after chemotherapy, followed by checkpoint immunotherapy.

The BMI of solid tumors is not as common as hematological tumors. Although it is reported to be up to 35% in patients with recurrent or metastatic tumors (4–8), the incidence will be lower in the real world because patients without bone aspiration were not included in the statistics.

A BMI in solid tumors is a special type of distant metastasis. Routine examinations of patients do not reveal bone marrow. The invasive bone marrow aspiration and biopsy, which is the only method of diagnosis, is not a routine examination for solid tumors. Positive biopsy results were defined as the presence of nonhematopoietic cell infiltration under a microscope. The clinical features and laboratory tests are not typical, making diagnosis difficult. The main clinical features of BMI are anemia, leukopenia, thrombocytopenia, bone pain, tenderness of the sternum, and fever. Laboratory tests show elevated LDH, ALP, GOT, and GPT levels (18, 19). In this case, the patient’s clinical features included anemia, bone pain, and fever. His laboratory tests showed elevated levels LDH, ALP, GOT, and GPT. BMI was promptly diagnosed and followed with antitumor therapy. The successful case revealed the importance of bone marrow biopsy, which should be considered a routine procedure in metastatic patients with BMI-relevant clinical features and laboratory tests.

Patients with BMI often have fatal complications that result in an even poorer prognosis (3, 18). A study of 83 cases of bone marrow metastatic solid tumors reported a median overall survival time of 49 days. In the 83 cases, anemia (95%) was the most common finding, followed by thrombocytopenia (77%), leukocytosis (31%), leukopenia (18%), and neutropenia (12%). Active bleeding was present in 18 of 83 patients (21%), and prolonged prothrombin times were detected in 33 of 67 patients (49%) (9). There is a lack of internationally recognized guidelines for the treatment of solid tumor patients with BMI. In general, chemotherapy-based systemic therapy is the preferred treatment for stage IV malignant tumors. However, patients with BMI often have poor general status and peripheral blood abnormalities. Because of its cytotoxicity, there is no consensus on whether BMI patients can benefit from chemotherapy-based systematic treatment. However, there is evidence that chemotherapy can still improve the overall prognosis, although more supportive treatments are required during the course, such as component transfusion, nutritional support, etc. (7, 20–22). Patients who have low KPS scores, infection susceptibility for leukocytopenia, bleeding susceptibility for thrombocytopenia, and DIC susceptibility for coagulation disorders have been unable to tolerate chemotherapy. Although it has not been discussed in previous articles, BMI patients without hemocytopenia may have a better prognosis.

The choice of chemotherapy regimens for solid tumors with BMI is quite individualized because ineffective chemotherapy may further aggravate the reduction in blood cells, which may shorten the survival time of patients. PDTXs have been shown to perform robustly as a translation platform for *in vivo* drug testing of efficacy (23, 24). In this case, a PDTX model was built before systemic therapy. *In vivo* experiments compared eight combinations of chemotherapy regimens. They are carboplatin plus paclitaxel, cisplatin plus paclitaxel, cisplatin plus docetaxel, cisplatin plus gemcitabine, cisplatin plus 5-fluorouracil plus docetaxel, cisplatin plus 5-fluorouracil plus cetuximab, cisplatin plus gemcitabine plus cetuximab, and cisplatin plus paclitaxel plus cetuximab. The tumor component, tumor cell necrosis ratio, and tumor cell proliferation index were used to assess the efficacy of the regimens. The toxicity of the regimens was also assessed based on the weight of the mice. As a result, the patient received the gemcitabine plus cisplatin plus cetuximab regimen, which demonstrated high efficacy and low toxicity. Cisplatin was not used in the first two cycles of chemotherapy because of the patient’s poor general condition.

Capecitabine and programmed death receptor 1 showed satisfactory curative effects as maintenance therapy in metastatic NPC patients (25, 26). In this case, the patient was diagnosed with dermatomyositis when he was initially diagnosed with NPC. As a kind of autoimmune disease, dermatomyositis may relapse and worsen under the action of programmed death receptor 1. Fortunately, this did not happen. Capecitabine plus sintilimab has been administered for over 8 months and counting. We found that the proportion of total peripheral T cells as well as the proportion of CD8^+^ T cells increased during the course of therapy. It may be related to the high efficiency and low toxicity of patients, which warrants further study.

There are two combinations of the regimens: gemcitabine plus cisplatin plus cetuximab and capecitabine plus sintilimab, which were successively conducted in the series of treatments for the patient. During the course of gemcitabine plus cisplatin plus cetuximab, the patient’s symptoms were relieved rapidly in the previous cycles of treatments, though cisplatin was not included in the first two cycles because of his poor general condition. He achieved continued remission in every two cycles of assessment. In addition, only suspicious tumor cells were detected in the bone marrow biopsy after six cycles of treatments. It shows that the regimen of gemcitabine plus cisplatin plus cetuximab plays a more critical role in this case.

This single-patient protocol for metastatic NPC with BMI with a significant response to cisplatin plus gemcitabine plus cetuximab followed by capecitabine plus sintilimab provides a new treatment option for this disease. The PDTX model is a feasible method of treatment prediction and formulation of individualized treatment for solid tumor patients with BMI. The analysis of total and proportion of peripheral T cells may predict patient’s treatment efficiency and toxicity, which warrants further study.

## Data Availability Statement

The original contributions presented in the study are included in the article/supplementary material. Further inquiries can be directed to the corresponding author.

## Ethics Statement

Written informed consent was obtained from the individual(s) for the publication of any potentially identifiable images or data included in this article.

## Author Contributions

QW and BZ contributed to the study concept and design. TZ contributed to the investigation and writing of the original draft. LJ contributed to the collection of biopsy data and analysis. YZ contributed to the collection of CT image data and analysis. All authors contributed to the article and approved the submitted version.

## Conflict of Interest

The authors declare that the research was conducted in the absence of any commercial or financial relationships that could be construed as a potential conflict of interest.

## Publisher’s Note

All claims expressed in this article are solely those of the authors and do not necessarily represent those of their affiliated organizations, or those of the publisher, the editors and the reviewers. Any product that may be evaluated in this article, or claim that may be made by its manufacturer, is not guaranteed or endorsed by the publisher.

## References

[1] Pasini F Pelosi G Mostacci R Santo A Cetto GL. Detection at Diagnosis of Tumor Cells in Bone Marrow Aspirates of Patients With Small-Cell Lung Cancer (SCLC) and Clinical Correlations. Ann Oncol (1995) 6(1):86–8. doi: 10.1093/oxfordjournals.annonc.a059056 7536032

[2] Ihde DC Simms EB Matthews MJ Cohen MH Minna JD. Bone Marrow Metastases in Small Cell Carcinoma of the Lung: Frequency, Description, and Influence on Chemotherapeutic Toxicity and Prognosis. Blood (1979) 53(4):677–86. doi: 10.1182/blood.V53.4.677.677 218597

[3] Bezwoda WR Lewis D Livini N. Bone Marrow Involvement in Anaplastic Small Cell Lung Cancer: Diagnosis, Hematologic Features, and Prognostic Implications. Cancer (1986) 58(8):1762–5. doi: 10.1002/1097-0142(19861015)58:8<1762::AID-CNCR2820580830>3.0.CO;2-V 3019512

[4] Cvitkovic E Bachouchi M Boussen H Busson P Rousselet G Mahjoubi R. Leukemoid Reaction, Bone Marrow Invasion, Fever of Unknown Origin, and Metastatic Pattern in the Natural History of Advanced Undifferentiated Carcinoma of Nasopharyngeal Type: A Review of 255 Consecutive Cases. J Clin Oncol (1993) 11(12):2434–42. doi: 10.1200/JCO.1993.11.12.2434 8246032

[5] Weiss L Grundmann E Torhorst J Hartveit F Harlos JP. Haematogenous Metastatic Patterns in Colonic Carcinoma: An Analysis of 1541 Necropsies. J Pathol (1986) 150(3):195–203. doi: 10.1002/path.1711500308 3806280

[6] Kamby C Guldhammer B Vejborg I Rossing N Dirksen H Daugaard S. The Presence of Tumor Cells in Bone Marrow at the Time of First Recurrence of Breast Cancer. Cancer (1987) 60(6):1306–12. doi: 10.1002/1097-0142(19870915)60:6<1306::AID-CNCR2820600624>3.0.CO;2-X 3621113

[7] Ingle JN Tormey DC Bull JM Simon RM. Bone Marrow Involvement in Breast Cancer. Effect on Response and Tolerance to Combination Chemotherapy. Cancer (1977) 39(1):104–11. doi: 10.1002/1097-0142(197701)39:1<104::AIDCNCR2820390119>3.0 832225

[8] Ruymann FB Newton WA Ragab AH Donaldson MH Foulkes M. Bone Marrow Metastases at Diagnosis in Children and Adolescents With Rhabdomyosarcoma a Report From the Intergroup Rhabdomyosarcoma Study. Cancer (1984) 53(2):369–73. doi: 10.1002/1097-0142(19840115)53:2<368::AID-CNCR2820530233>3.0.CO;2-3 6546301

[9] Hung YS Chou WC Chen TD Chen TC Chen JS. Prognostic Factors in Adult Patients With Solid Cancers and Bone Marrow Metastases. Asian Pacific J Cancer Prev (2014) 15(1):61–7. doi: 10.7314/APJCP.2014.15.1.61 24528082

[10] Rajagopalan V Kamar F Thayaparan R Grossbard ML. Bone Marrow Metastases From Glioblastoma Multiforme – a Case Report and Review of the Literature. J Neuro-Oncol (2005) 72(2):157–61. doi: 10.1007/s11060-004-3346-y 15925996

[11] Jani P Charles CY. Massive Bone Marrow Involvement by Clear Cell Variant of Rhabdomyosarcoma. Indian J Pediatr (2009) 76(2):224–8. doi: 10.1007/s12098-008-0230-3 19129993

[12] Rossi A Colantuoni G Cantore N Panico L Gridelli C. Complete Response of Severe Symptomatic Bone Marrow Metastases From Heavily Pretreated Breast Cancer With a 3-Weekly Trastuzumab Schedule. A Clinical Case. Anticancer Res (2004) 24(1):317–20. doi: 10.1016/j.jneumeth.2011.07.009 15015614

[13] Saito M Kiyozaki H Chiba F Takata O Yoshida T Shuto C. Early Gastric Cancer Combined With Multiple Metachronous Osteosclerotic Bone and Bone Marrow Metastases That Responded to Chemoradiotherapy. Gastric Cancer (2011) 14(3):295–9. doi: 10.1007/s10120-011-0062-0 21671047

[14] Artac M Koral L Toy H Guler T Boruban MC Altundag K. Complete Response and Long-Term Remission to Anti-Her2 Combined Therapy in a Patient With Breast Cancer Presented With Bone Marrow Metastases. J Oncol Pharm Pract (2014) 20(2):141–5. doi: 10.1177/1078155213480201 23676508

[15] Nakamura S Fukui T Suzuki S Takeda H Watanabe K Yoshioka T. Long-Term Survival After a Favorable Response to Anti-EGFR Antibody Plus Chemotherapy to Treat Bone Marrow Metastasis: A Case Report of KRAS-Wildtype Rectal Cancer. OncoTarg Ther (2017) 10:1143. doi: 10.2147/OTT.S129275 PMC5328292 28260928

[16] Berry S Mallik T Rapado F Morar P. Bone Marrow Metastasis in Primary Nasopharyngeal Carcinoma. Otolaryngol Head Neck Surg (2006) 135:163–4. doi: 10.1016/j.otohns.2005.03.046 16815206

[17] Miyaushiro S Kitanaka A Kubuki Y Hidaka T Shide K Kameda T. Case Report Nasopharyngeal Carcinoma With Bone Marrow Metastasis: Positive Response to Weekly Paclitaxel Chemotherapy. Internal Med (2015) 54:1455–9. doi: 10.2169/internalmedicine.54.3917 26028007

[18] Tritz DB Doll DC Ringenberg QS Anderson S Madsen R Perry MC. Bone Marrow Involvement in Small Lung Cancer. Clinical Significance and Correlation With Routine Laboratory Variables. Cancer (1989) 63(4):763–6. doi: 10.1002/1097-0142(19890215)63:43.0.CO;2-F 2536586

[19] Chernow B Wallner SF. Variables Predictive of Bone Marrow Metastasis. Cancer (1978) 42(5):2373–8. doi: 10.1002/1097-0142(197811)42:5<2373::AID-CNCR2820420538>3.0.CO;2-P 719614

[20] Rodriguez-Kraul R Hortobagyi GN Buzdar AU Blumenschein GR. Combination Chemotherapy for Breast Cancer Metastatic to Bone Marrow. Cancer (1981) 48(2):227–32. doi: 10.1002/1097-0142(19810715)48:2<227::AID-CNCR2820480203>3.0.CO;2-M 7237395

[21] Kopp H Krauss K Fehm T Staebler A Zahm J Kanzet L. Symptomatic Bone Marrow Involvement in Breast Cancer - Clinical Presentation, Treatment, and Prognosis: A Single Institution Review of 22 Cases. Anticancer Res (2011) 31(11):4025–30. doi: 10.1093/hsw/33.2.87 22110237

[22] Wong BL Gan RWC Adabavazeh B Jose J. Bone Marrow Metastasis/Carcinomatosis in Head and Neck Squamous Cell Carcinoma. J Oral Pathol Med (2021) 00:1–7. doi: 10.1111/jop.13184 33818835

[23] Georgopoulou D Callari M Rueda OM Shea A Caldas C. Landscapes of Cellular Phenotypic Diversity in Breast Cancer Xenografts and Their Impact on Drug Response. Nat Commun (2021) 12(1):1998. doi: 10.1038/s41467-021-22303-z 33790302 PMC8012607

[24] Zhang J Zhang L Su X Li M Xie L Malchers F. Translating the Therapeutic Potential of Azd4547 in Fgfr1-Amplified Non-Small Cell Lung Cancer Through the Use of Patient-Derived Tumor Xenograft Models. Clin Cancer Res (2012) 18(24):1–10. doi: 10.1158/1078-0432.CCR-12-2694 23082000

[25] Yang Y Qu S Li J Hu C Xu M Li W. Camrelizumab Versus Placebo in Combination With Gemcitabine and Cisplatin as First-Line Treatment for Recurrent or Metastatic Nasopharyngeal Carcinoma (CAPTAIN-1st): A Multicentre, Randomised, Double-Blind, Phase 3 Trial. Lancet Oncol (2021) 22:1162–74. doi: 10.1016/S1470-2045(21)00302-8 34174189

[26] Sun X Liu S Liang Y Chen Q Li X Tang L. The Role of Capecitabine as Maintenance Therapy in *De Novo* Metastatic Nasopharyngeal Carcinoma: A Propensity Score Matching Study. Cancer Commun (2020) 40(1):1–11. doi: 10.1002/cac2.12004 PMC7163789 32112522

